# Synthesis and Characterization of Porous Forsterite Ceramics with Prospective Tissue Engineering Applications

**DOI:** 10.3390/ma15196942

**Published:** 2022-10-06

**Authors:** Andrada Elena Alecu, Gabriel-Costin Balaceanu, Adrian Ionut Nicoara, Ionela Andreea Neacsu, Cristina Busuioc

**Affiliations:** 1 Department of Science and Engineering of Oxide Materials and Nanomaterials, Faculty of Chemical Engineering and Biotechnologies, University Politehnica of Bucharest, 011061 Bucharest, Romania; 2 National Research Center for Micro and Nanomaterials, Faculty of Chemical Engineering and Biotechnologies, University Politehnica of Bucharest, University Politehnica of Bucharest, 060042 Bucharest, Romania

**Keywords:** porous scaffolds, forsterite, sucrose, bone regeneration

## Abstract

Due to the urgent need to develop and improve biomaterials, the present article proposes a new strategy to obtain porous scaffolds based on forsterite (Mg_2_SiO_4_) for bone tissue regeneration. The main objective is to restore and improve bone function, providing a stable environment for regeneration. The usage of magnesium silicate relies on its mechanical properties being superior to hydroxyapatite and, in general, to calcium phosphates, as well as its high biocompatibility, and antibacterial properties. Mg_2_SiO_4_ powder was obtained using the sol-gel method, which was calcinated at 800 °C for 2 h; then, part of the powder was further used to make porous ceramics by mixing it with a porogenic agent (e.g., sucrose). The raw ceramic bodies were subjected to two sintering treatments, at 1250 or 1320 °C, and the characterization results were discussed comparatively. The porogenic agent did not influence the identified phases or the samples’ crystallinity and was efficiently removed during the heat treatment. Moreover, the effect of the porogenic agent no longer seems significant after sintering at 1250 °C; the difference in porosity between the two ceramics was negligible. When analysing the in vitro cytotoxicity of the samples, the ones that were porous and treated at 1320 °C showed slightly better cell viability, with the cells appearing to adhere more easily to their surface**.**

## 1. Introduction

Bone tissue has an increased self-regeneration capacity; small fractures heal naturally over time. However, serious lesions, with an average area above a critical size (around 2 cm^2^, depending on the anatomical site), will not regenerate without help [1]. Traumatic injuries, degenerative diseases, histological diseases, birth defects, or the removal of certain tumors can cause serious damage to bone tissue, which requires surgery to restore function and complete healing. The study of bone tissue regeneration has been and still is a major point of interest in the scientific world. At this intersection, materials science, mechanical engineering, electronics, computer science, medicine, and genetics collaboratively aim to improve existing methods and develop new methods of bone healing. Based on recent studies, the prevalence of bone diseases is increasing, whether related to body self-induced diseases or as a result of various external factors [2,3]. This has started to also affect the middle-aged population, not just the elderly, for which these diseases are inevitable, taking into account human nature.

Conventional treatment techniques from traditional medicine prove to be ineffective in several cases; therefore, they can be further enhanced. Moreover, due to the high need to understand, propose and develop new strategies that reach beyond the current level of knowledge and its efficiency, new methods have been proposed by the scientific community. Tissue engineering based on scaffolds has great potential in this respect. Scaffolds are 3D porous structures or hydrogels designed to define and support the development of new tissue, providing mechanical sustainability and providing certain factors locally to enhance growth [4,5]. The main objective of this concept is to restore and improve bone function, providing a stable environment for regeneration. Researchers in this field focus on developing and improving materials that mimic the endemic biological environment as much as possible. For this to be possible, certain properties of bone tissue (architecture, porosity, mechanical strength, cell adhesion, biocompatibility, cell proliferation, mineralization, and osteogenic differentiation) must be taken into account and then mimicked in the prototype of the scaffold [6,7].

Numerous scaffolds, produced from a variety of biomaterials and by various manufacturing techniques, have been used to regenerate tissues and organs. Among these materials are: tricalcium phosphates (TCP) [8,9,10,11], hydroxyapatite (HAp) [12,13,14], calcium carbonates [15], silicates [16,17,18], poly-D-lactide, poly-L-lactide (PLA) [19], poly-lactide-co-glycolide (PLGA) [20], poly-caprolactone [21], collagen [22,23], chitosan [24], gelatine [25], etc. HAp has been extensively used as bioceramic in bone tissue regeneration; however, alternative materials are currently being researched because of its poor mechanical resistance, which limits its applications.

Forsterite is a magnesium orthosilicate, a member of the olivine solid solution series [26,27]. Forsterite may be a bioactive ceramic with prospective applications in bone regeneration due to its biocompatibility, bioactivity, good degradation rate, increased solubility [28], very good mechanical properties [29], and low production costs [30]. Most of these properties can be attributed to Si and Mg ions. Studies have shown that the Si ion has a beneficial effect on bone metabolic processes and calcification [31,32]. Specifically, Si influences the production of type I collagen, improves bioactivity, promotes the differentiation of osteoblasts, and enhances bone mineralization [28]. Additionally, the Mg^2+^ ion is one of the most important elements in the human body, being responsible, among other things, for the mineralization of bones, their fragility, and also cell viability, contributing to the formation of osteoblasts [33,34]. Mg is the main substitute for Ca in biological apatite [35]. Forsterite has superior mechanical properties to HAp and, in general, to calcium phosphates [34]. Highly porous forsterite nanostructured scaffolds with high compressive strength, as well as good bioactivity and degradability were prepared by conducting a two-step sintering method; it was found that the formation of glassy enstatite is responsible for increasing the mechanical behaviour [36]. A multi-step sintering method was also applied to nanocrystalline forsterite powder to produce highly porous scaffolds with the foamy method; the results demonstrated their suitability for load-bearing applications, in the range of cancellous bone [37]. Zhu et al. [38], fabricated porous forsterite scaffolds by combining a 3D printing and polymer-derived ceramics strategy, which possess photothermal-induced antibacterial activity, high compressive strength and a low degradation rate. Other researchers [39] manufactured a nanostructured forsterite scaffold via the gel casting method and coated it with sol-gel derived bioactive glass in order to achieve good mechanical support and remarkable bioactivity. Aghajanian et al. [40], employed a forsterite/poly-3-hydroxybutyrate composite as coating for a porous titanium scaffold, achieving a higher apatite forming ability compared to the uncoated one and a significant increase in cell viability. Hierarchical scaffolds based on poly(lactic acid) microstructures and nanocomposite gelatin-forsterite fibrous layers were developed using fused deposition modelling and electrospinning, which led to appropriate mechanical and biological properties for developing bone substitutes [41].

In recent years, specialized studies have highlighted the biocompatible and antibacterial effect of forsterite ceramics [42], which makes this material sustainable for the development of bone grafts. Taking into account the characteristics mentioned above regarding forsterite, the present study aims to develop ceramics with controlled porosity by adding porogenic materials such as sucrose. Additionally, a morpho-structural characterization of these ceramics is performed, as well as in vitro characterization of their biocompatibility.

## 2. Materials and Methods

### 2.1. Materials

To obtain forsterite, the raw materials used were magnesium nitrate hexahydrate—Mg(NO_3_)_2_∙6H_2_O (Sigma-Aldrich, BioXtra, *p* ≥ 98%, Darmstadt, Germany), tetraethyl orthosilicate—(C_2_H_5_O)_4_Si (Sigma-Aldrich, reagent grade, *p* ≥ 98%, Darmstadt, Germany), ethanol (Sigma-Aldrich, *p* ≥ 99.8%, Darmstadt, Germany) and distilled water.

First, Mg(NO_3_)_2_∙6H_2_O was dissolved in 150 mL ethanol under magnetic stirring for 20 min at 60 °C. After 20 min, the required amount of TEOS (Tetraethyl orthosilicate, Sigma-Aldrich, Darmstadt, Germany) to keep Mg:Si molar ratio of 2:1, was added dropwise. To dissolve all precursors, the mixture was continuously magnetically stirred for an additional hour. After homogenization, the reaction mixture was placed in the oven (Electro-Total, Bucharest, Romania) at 60 °C for the gelation process. The resulting gel underwent a drying process in the oven, at 80 °C, until all the liquid was eliminated. After drying, the mixture was ground into a fine powder and sieved, to homogenize the obtained powder. Once the fine, homogenous forsterite powder was obtained, it was calcinated at 800 °C for 2 h.

Part of the previously calcined forsterite was further used to make a porous ceramic, by mixing it with 20%wt. commercial sucrose (Sigma-Aldrich, ≥99.5%, Darmstadt, Germany) as a porogenic agent. Thus, after applying the required amount of glucose, the powders were ground and homogenized in a mortar and then pressed using a uniaxial press (pressure of 15 MPa).

In the specialized literature, there are several experimental studies that place the optimal temperature for obtaining forsterite above the temperature of 1200 °C. At lower temperatures, a large amount of secondary phase is obtained. Increasing the sintering temperatures above this minimum (in the range of 1350–1550 °C) leads to obtaining pure forsterite phase; however, the required sintering time exceeds 3 h. Additionally, increasing the sintering temperature increases the mechanical resistance of the obtained ceramic bodies [43,44,45].

The aim of the current work is to obtain the purest forsterite bioceramic, with the lowest possible energy cost and with a mechanical resistance as close as possible to that of natural bones. Therefore, from the sintering intervals reported in the literature, two intermediate temperatures were chosen and it was obtained for a shorter sintering time of only 2 h.

After compacting the powders into cylindrical raw bodies, they were subjected to two sintering treatments, at 1250 °C or 1320 °C, and compared. In all cases, the sintering time was 2 h and the step of the thermal process was 10 °C/min. More information regarding the resulting sintered ceramic bodies can be found in Table 1.

### 2.2. Structural and Morphological Characterization

The X-ray diffraction (XRD) technique was used to determine the degree of crystallinity, the crystallite size, the crystallization type and to identify the phases present in the samples. The analysis was carried out using a PANalytical Empyrean diffractometer (Malvern Panalytical, Almelo, The Netherlands) at room temperature, with a characteristic Cu X-ray tube (λ CuKα = 1.541874 Å). The samples were scanned in a Bragg–Brentano geometry, with a scan step increment of 0.02° and a counting time of 100 s/step. The XRD patterns were recorded in the 2θ angle range of 10–80°. Morphological aspects of the obtained ceramic materials were studied via Scanning electron microscopy (SEM), with a Quanta Inspect F50 microscope coupled with an X-ray energy dispersion spectrometer (EDS) (Thermo Fisher, Eindhoven, Netherland). The pore size distribution was determined by measuring the pore size using ImageJ 1.50i software (Wayne Rasband National Institute of Health, 2016, MD, USA) and the obtained data were graphically represented as a histogram using OriginPro 9.0 software (OriginLab, 2012, Northampton, MA, USA). Data are presented as mean ± standard error.

The mechanical compression strength was determined using a cylindrical sample (φ = 13 mm, h = 2.5 mm) with Shimadzu Autograph AGS-X 20kN (Shimadzu, Tokyo, Japan) equipment. To obtain more accurate results, all determinations were performed in triplicate at the speed 0.5 mm/min. The investigation with the Fourier transform infrared spectroscopy (FT-IR) method of the synthesized ceramic bodies was performed using a Nicolet iS50R spectrometer (Thermo Fisher, Waltham, MA, USA). The measurements were made at room temperature, using the total reflection attenuation module. Each sample was scanned 32 times between 4000 and 400 cm^−1^, at a resolution of 4 cm^−1^.

The ceramic properties (absorption capacity and open porosity) of the obtained materials were evaluated using the Archimedes method and calculated using Equations (1)–(3). Specifically, the samples are initially weighed in air and placed in a vacuum desiccator. After vacuuming for 15 min (time required to remove the air from the pores), the samples are immersed in a liquid. It is recommended that a liquid with a density lower than the density of the ceramic body be used so that it can be completely covered by the liquid. In this particular study, distilled water was used to penetrate the pores of the samples. Subsequently, the samples saturated in water are pulled off in a humid cotton cloth to remove the liquid from the surface, then weighed again, both in air and then water.

(1) $$\rho_{a}=\frac{m_{i}\cdot \rho_{x}}{m_{xa}-m_{x}}\cdot \frac{g}{cm^{3}}$$

(2) $$A=\frac{\left(m_{xa}-m_{i}\right)}{m_{xa}}\cdot 100\%$$

(3) $$P=\frac{\left(m_{xa}-m_{i}\right)}{\left(m_{xa}-m_{x}\right)}\cdot 100\%$$

where *m_i_*—the initial weight of the sample (g); *m_xa_*—the weight (measured in air medium) of the liquid impregnated sample (g); *m_x_*—the weight (measured in ethanol medium) of the liquid impregnated sample (g); *ρ_a_*—apparent density (g/cm^3^); *ρ_x_*—water density (1 g/cm^3^); *A*—absorption capacity (%); *P*—open porosity (%).

The thermal shrinkage was calculated by measuring the cylindrical samples using a caliper. The measurements were performed before and after the sintering process so that the assessment shrinkage could be performed as a function of volume (V). Height (h) and diameter (d) were measured and then equations (4) and (5) were applied. The weight loss was similarly measured, taking into account the weight of the sample before (m_0_) and after sintering (m_1_).

(4) $$\Delta m=\frac{\left(m_{0}-m_{1}\right)}{m_{0}}\cdot 100\%$$

(5) $$C=\frac{\left(V_{f}-V_{i}\right)}{V_{i}}\cdot 100\%$$

where *m_0_*—the weight of the sample before sintering (g); *m_1_*—the weight of the sample after sintering (g); *V_i_*—the volume of the sample before sintering (cm^3^); *V_f_*—the volume of the sample after sintering (cm^3^).

All tests were performed in triplicate for each ceramic obtained and data are presented as means values ± standard error from the triplicate analysis.

### 2.3. Biomineralization Capacity and In Vitro Cytotoxicity Assays

The biomineralization capacity was evaluated by immersing the ceramic bodies in SBF (simulated body fluid) solution at 37 °C for 14 days. The preparation of the SBF solution was performed by following Kokubo’s recipe and standard procedure [46].

The determination of cell viability was performed in triplicate using the Live/Dead Viability/Cytotoxicity Kit (Thermo Scientific, Waltham, MA, USA). The images were obtained with the LSM 880 confocal microscope (Carl Zeiss, Göttingen, Germany).

First, a series of amniotic fluid stem cells (AFSC) purchased from the Coriell Institute (Kenton, NJ, USA) were cultivated inside 35 mm disposable petri dishes for 3 days until acceptable cell densities were obtained. The synthesized materials were added in the respective culture cell in which the fluorophores provided by the Live/Dead Viability Kit were added. After 24 h from inoculation, samples were prepared with fluorescence microscopy. This preparation aimed to introduce fluorophores into the ceramic materials tested so that living and dead cells that adhered to the material surface could be highlighted.

The specific fluorophores were Calcein (Sigma-Aldrich, ≥95.0%, Darmstadt, Germany), a specific polyanionic dye for the detection of living cells (by producing an intense green fluorescence on microscopic evaluation) and Ethidium Homodimer-1 (Sigma-Aldrich, Darmstadt, Germany) for the detection of dead cells (by binding to the nucleic acids of the cells, a red colour is produced).

## 3. Results and Discussions

**Figure 1:**
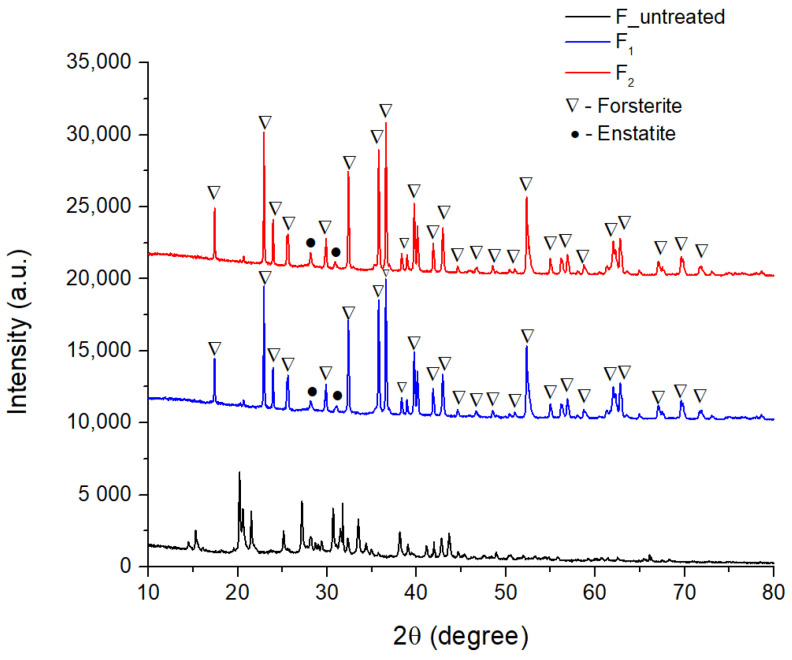
X-ray patterns of forsterite samples: untreated, F_1_ and F_2_.

Figure 1 shows the X-ray patterns for the untreated forsterite, as well for the F_1_, F_2_ samples. In the case of the calcined sample, a lower intensity of the diffraction interferences could be observed, which suggests that the phases characteristic of forsterite were not yet formed. A small full width at the half maximum (FHWM) parameter was registered, which means that the crystallites dimension was slightly larger. When the temperature increased to within the specific interval of forsterite phase crystallization, the presence of diffraction interferences with high intensities that were well-defined were obvious, which were attributed to the orthorhombic forsterite phase (Mg_2_SiO_4_ identified by ICDD 01-080-0783). It should be noted that the emergence of a second phase is observed (monoclinic enstatite—MgSiO_3_, identified by ICDD 00-019-0769), but the diffraction indices are very low in intensity. The most intense diffraction index is specific to the diffraction angle 2θ = 36.5°.

**Figure 2:**
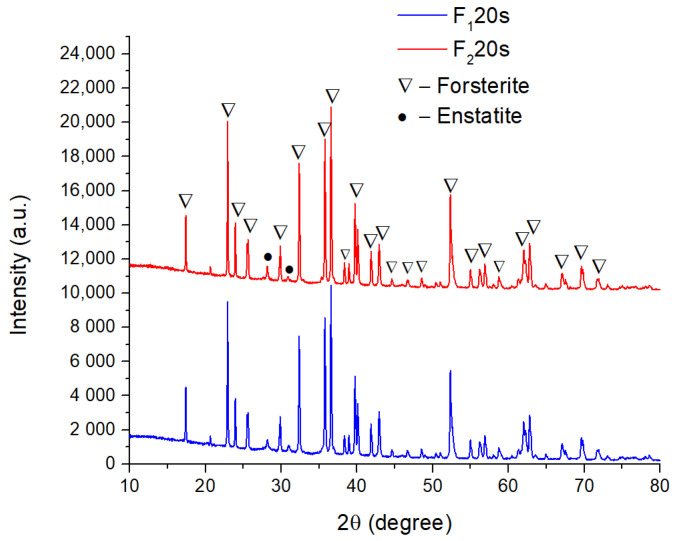
X-ray patterns of forsterite samples: F_1_20s and F_2_20s.

Figure 2, corresponding to the sintered ceramic bodies with sucrose, indicates that the porogenic agent did not influence the identified phases or the crystallinity of the samples, and was efficiently removed from the samples during heat treatment.

**Figure 3:**
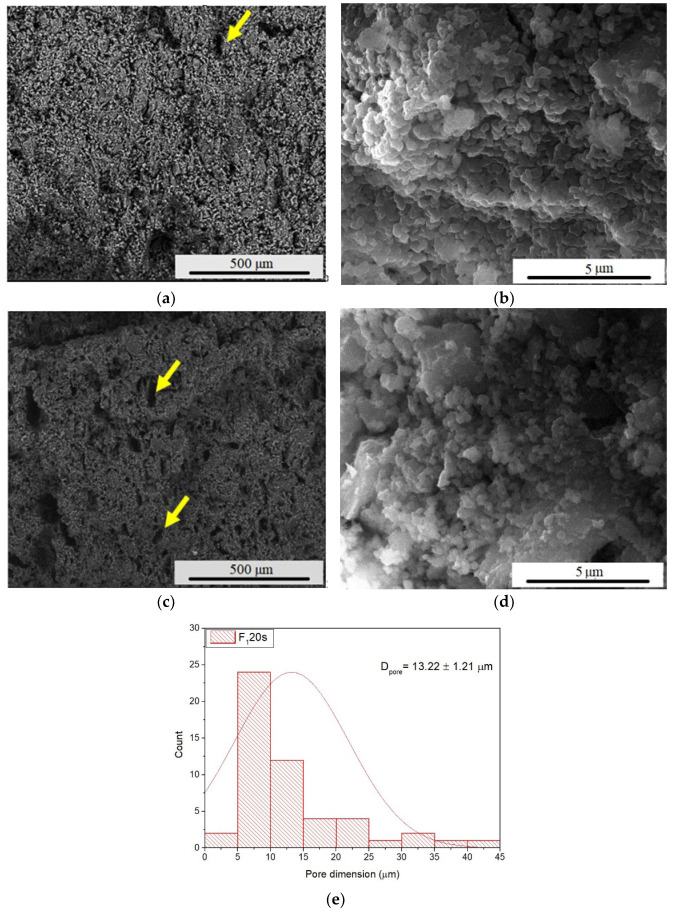
SEM images magnification ×200 and ×20,000 of F_1_ (**a**,**b**) and F_1_20s (**c**,**d**) samples and pore dimension distribution (**e**); the pores are indicated with yellow arrows.

Figure 3 presents the influence of the porogenic agent on the microstructure of the samples, evaluated by SEM (scanning electron microscopy). Hence, a predominantly spherical morphology of the particles was identified, with the presence of certain irregular polyhedral particles for the F_1_ and F_1_20s samples, respectively. The average particle size is 91 nm. The porogenic agent influenced the porosity of samples, without altering the morphology of forsterite crystals, as well as the particle size.

When sintering forsterite ceramic bodies (sample F_1_) at 1250 °C, the presence of pores is visible (yellow arrow), but they are in a relatively small number. When forsterite powder was mixed with a porogenic agent of 20% wt., a higher number of pores with average dimensions of 13.22 μm was found (sample F_1_20s—see Figure 3e).

**Figure 4:**
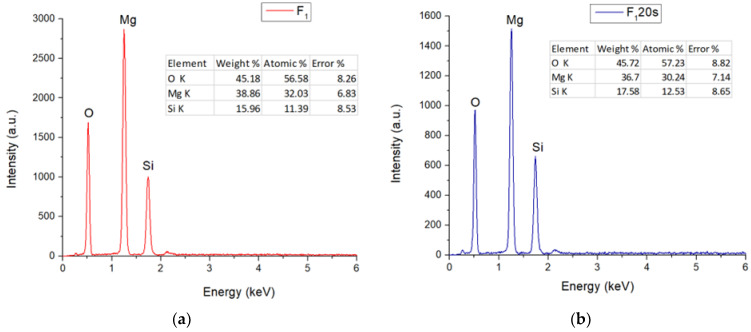
EDS spectra for: (**a**) F_1_; (**b**) F_1_20s samples.

Figure 4 exhibits the EDS (X-ray energy dispersion) spectra of the F_1_ and F_1_20s samples. It can be seen that, in both cases, the specific elements of forsterite (Mg, Si, O) were identified, once again confirming that the elements present in the porogenic agent did not remain in the sample composition, influencing only its porosity.

**Figure 5:**
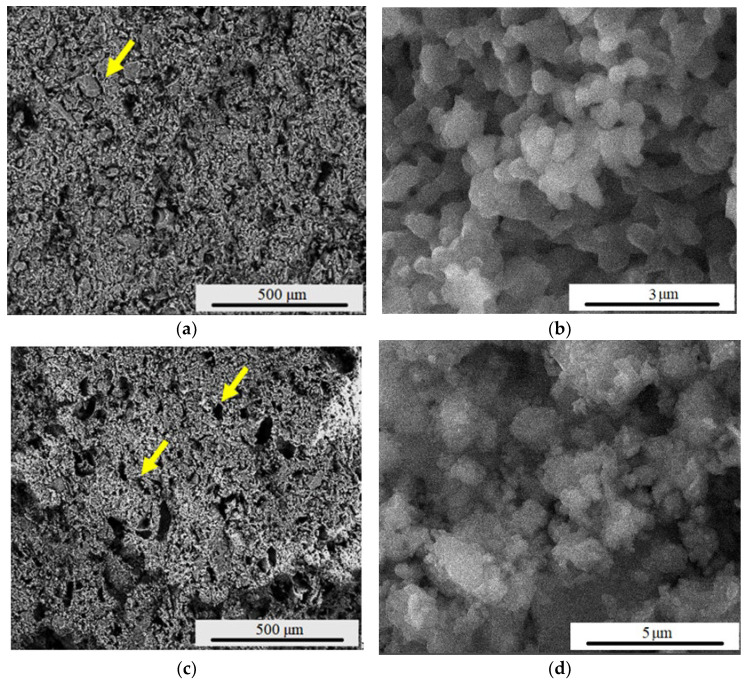

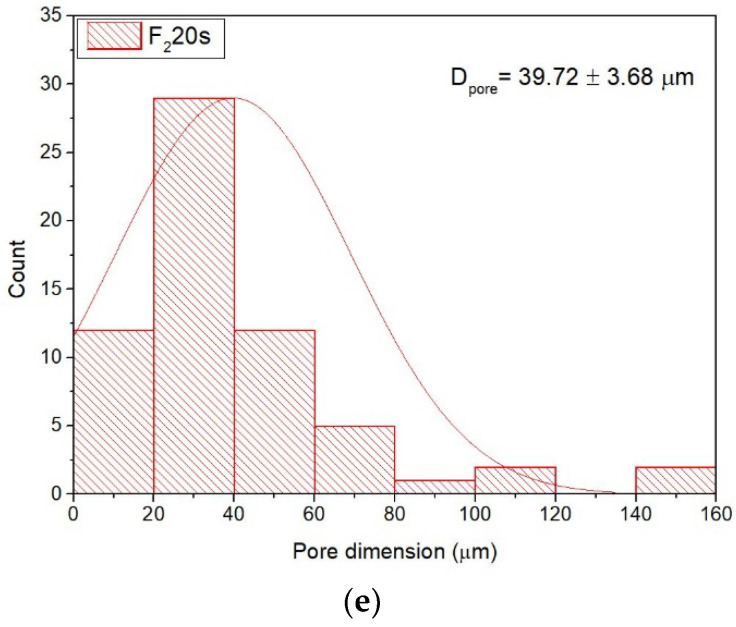
SEM images magnification ×200 and ×20,000 of F_2_ (**a**,**b**) and F_2_20s (**c**,**d**) samples and pore dimension distribution (**e**); the pores are indicated with yellow arrows.

Figure 5 presents the influence of the sintering temperature on the microstructure of the samples, evaluated by SEM. Thus, Figure 5a,b presents the surface morphology of the F_2_ sample, having mostly spherical particles, as well as pores. The average particle size, in this case, is around 300 nm (increase most likely due to particle agglutination). Regarding the F_2_20s sample (Figure 5c,d), in which a porogenic agent was introduced before the sintering process, a higher number of pores with average dimensions of 39.72 μm are present in the sample structure (see yellow arrow).

**Figure 6:**
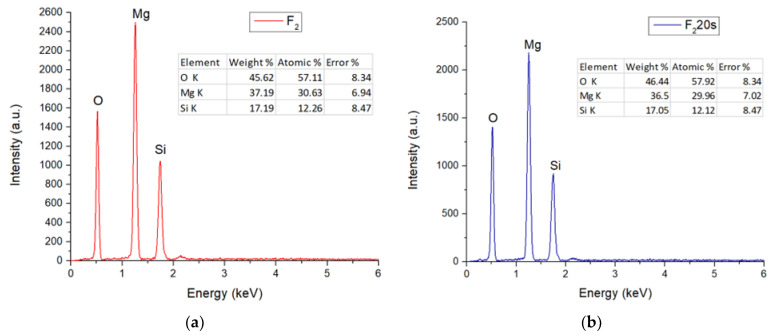
EDS spectra for F_2_ (**a**) and F_2_20s (**b**) samples.

The EDS spectra (Figure 6) indicate that the porogenic agent did not influence the chemical composition of the samples, identifying only the presence of the characteristic elements of forsterite (Mg, Si, O).

**Figure 7:**
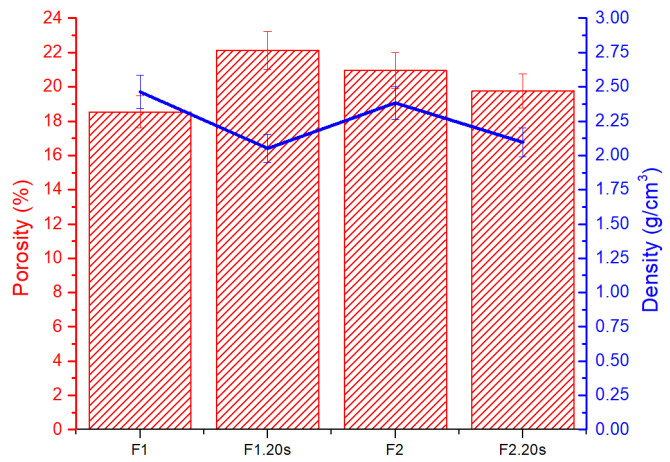
Porosity and density vs. temperature for all forsterite samples.

Figure 7 shows the porosity of forsterite samples at different treatment temperatures. It can be observed that in the case of forsterite treated at 1250 °C, the porosity is significantly lower (18.53%) than in the case of forsterite in which porogenic agent was added and treated at 1250 °C (22.11%). Increasing the temperature at 1320 °C, the porosity (20.95%) is found to be close to that of the forsterite sample with porogenic agent treated at 1250 °C. However, it seems that with the increase in temperature, the effect of the porogenic agent is no longer significant; the sample of forsterite from 1320 °C in which sucrose was added does not present a better porosity than in the case of the sample from 1320 °C without the porogenic agent. This can be explained by the increase in the specific surface of the samples and the agglutination of their particles at a temperature of 1320 °C, restricting the pore space. Comparing the obtained results with the bone tissue porosity, it can be stated that forsterite falls within the specific porosity range of the cortical bone (5–40%) [47,48].

**Figure 8:**
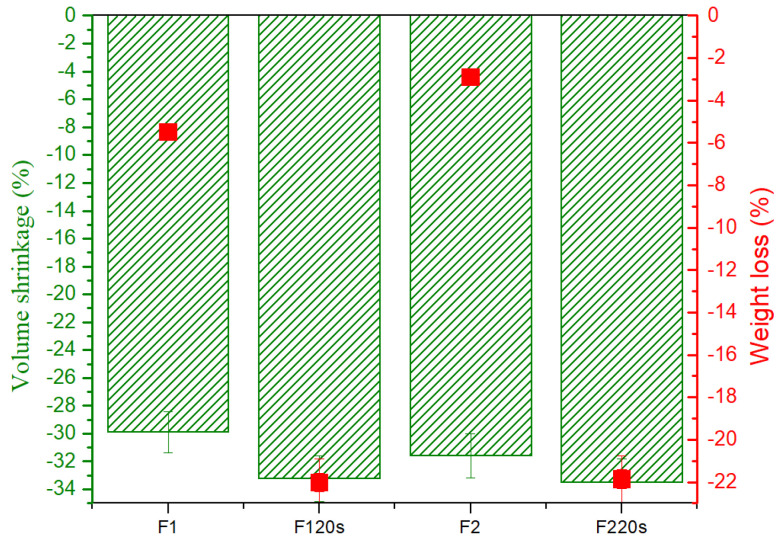
The effect of heat treatment on shrinkage and weight loss of all forsterite samples.

Figure 8 shows the shrinkage and weight loss of forsterite samples as a function of working temperatures. It is obvious that in the case of samples F_1_20s and F_2_20s, the combustion contraction (33.23 and 33.49%, respectively) was superior to samples F_1_ and F_2_ (29.88 and 31.58%, respectively) with the exposure to heat treatments. Additionally, the weight loss is more pronounced in the case of samples in which the porogenic agent was introduced. The explanation for these behaviours is found in the fact that sucrose was removed through the heat treatments to which the samples were subjected.

**Figure 9:**
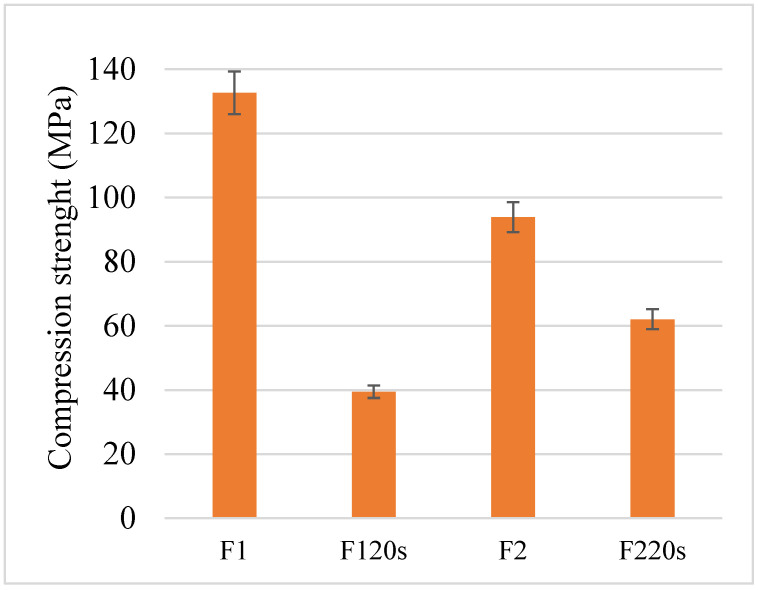
The compression strength of all forsterite samples.

The samples’ compressive strength is presented in Figure 9. It can be seen that the F_1_20s sample, which showed the highest porosity, is also the most prone to compressive load failure, becoming deformed at 40 MPa. This confirms the already known results in the literature according to which the material resistances are affected by the increase in its porosity. In the case of forsterite, the temperature negatively influenced the compressive strength. Thus, the F_2_ sample, which was treated at a temperature of 1320 °C, showed a lower compressive strength of 94 MPa than the F_1_ sample treated at a lower temperature. The most resistant sample after the tests was F_1_, with a very good resistance of around 132 MPa. This falls within the range of compressive strengths of cortical bone tissue, far exceeding the resistance of the trabecular bone [42] and being another favourable point in terms of optimal treatment temperature of forsterite between 1200 and 1300 °C. F_2_20s withstood a pressure up to 62 MPa.

According to Choudhary et al. [42], the range of the compressive strength of trabecular bone tissue is between 0.1 and 16 MPa, and for cortical bone tissue it is between 130 and 200 MPa. These results demonstrate that the F_1_ sample is suitable for both bone types, while the F_2_ sample is also close to the standard of cortical bone tissue.

**Figure 10:**
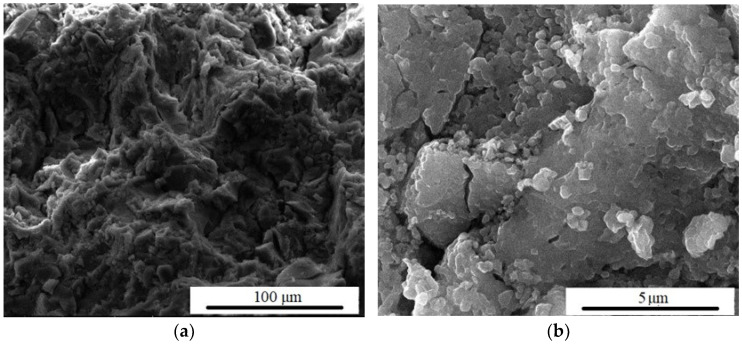

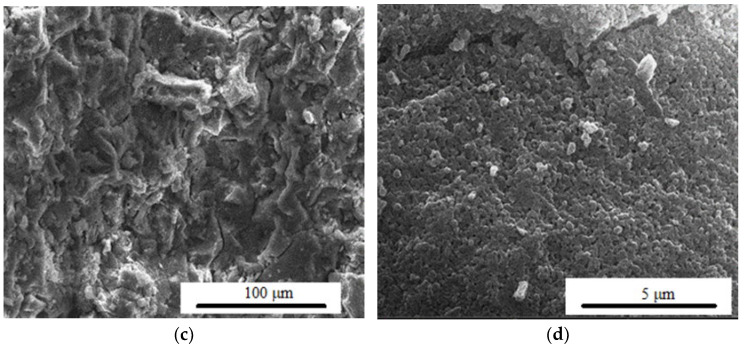
SEM images magnification ×1000 and ×20,000 of F1 (**a**,**b**) and F120s (**c**,**d**) samples, after 14 days in SBF (simulated body fluid).

Figure 10 shows the SEM analysis of F_1_ and F_1_20s samples after immersion in SBF. The obvious decrease in pore size suggests that the HAp layer managed to grow inside them, thus mineralizing the surface. In Figure 10b,d, it can be seen how the samples’ surface tends to become more compact, which may be explained by the development of HAp aggregates. This can be seen both at magnifications of ×1000 (100 µm) and when the magnifications are increased to ×20,000 (5 µm).

**Figure 11:**
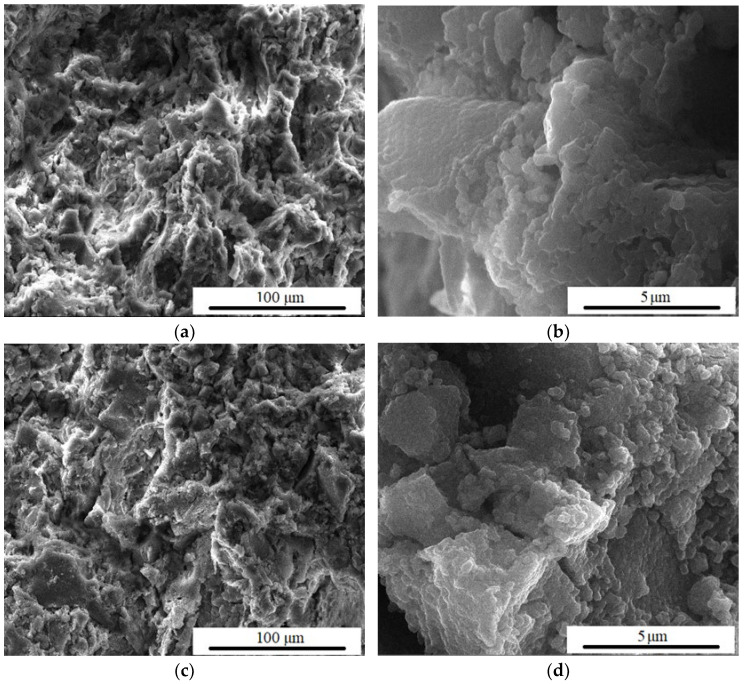
SEM images magnification ×1000 and ×20,000 of F_2_ (**a**,**b**) and F_2_20s (**c**,**d**) samples, after 14 days in SBF.

In Figure 11, where F_2_ and F_2_20s samples are found, the same phenomena can be seen as in the previous case, namely the pores seem to close and the surface becomes denser. These aspects can be explained by the deposition of HAp particles.

**Figure 12:**
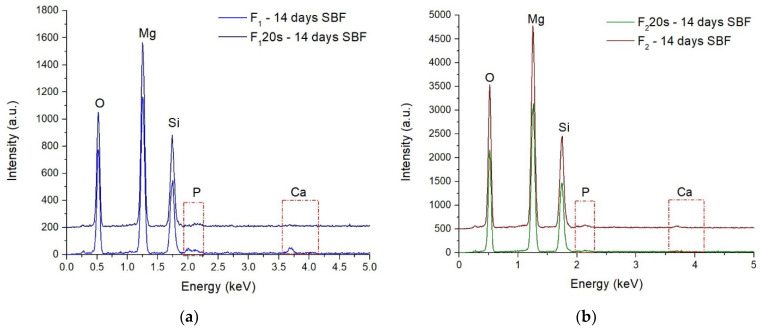
EDS spectra for F_1_, F_1_20s samples (**a**) and F_2_, F_2_20s samples (**b**) after 14 days in SBF.

The EDS spectra (Figure 12) and chemical composition (Table 2) performed on all samples indicate the presence of the Ca and P characteristic of the early formation of the apatite phase on the forsterite surface.

**Figure 13:**
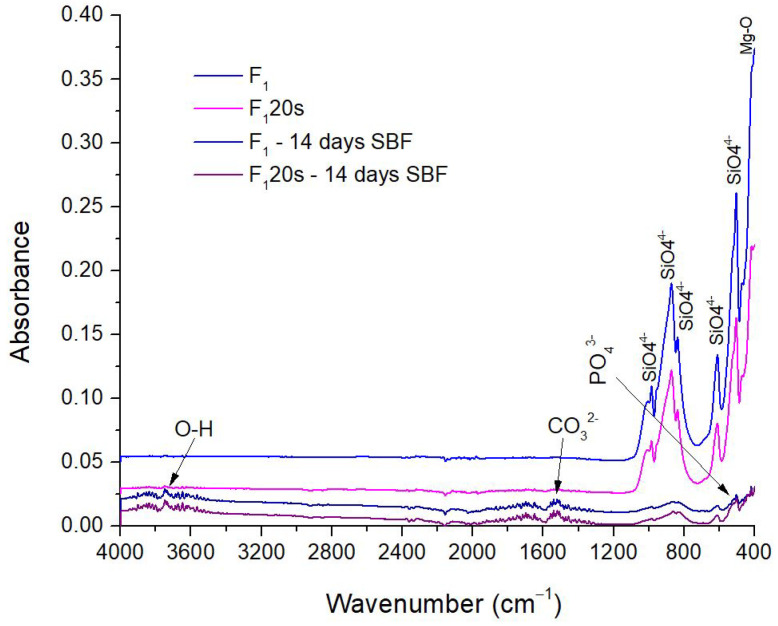
FTIR Spectra of F_1_, F_1_20s before and after 14 days in SBF.

**Figure 14:**
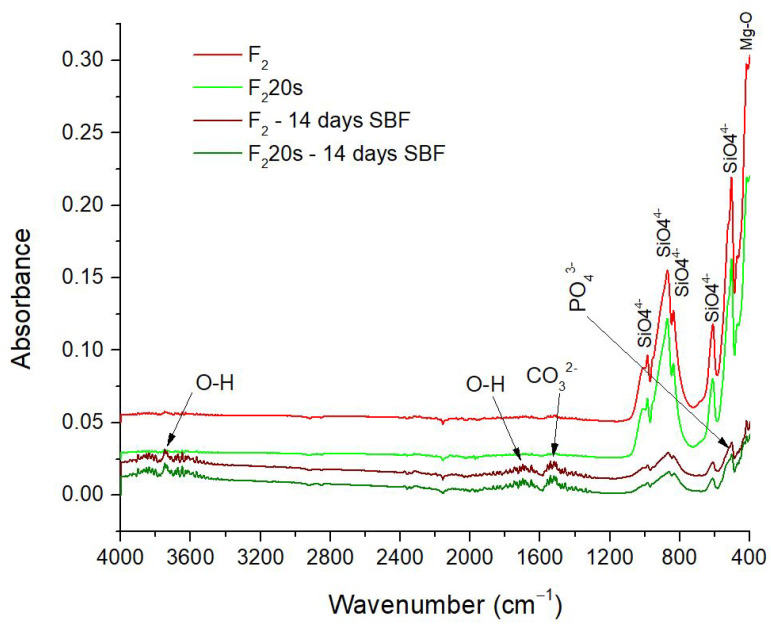
FTIR Spectra of F_2_, F_2_20s before and after 14 days in SBF.

Figure 13 and Figure 14 show the specific bands of hydroxyapatite that adhered to forsterite. In addition to the main bonds in the forsterite sample, the presence of OH bonds can be observed as specific vibrations bands situated at 1700 cm^−1^ and in the range of 3500–3900 cm^−1^. The carbonate groups are also present, the stretching vibrations are found in the range of 1500–1555 cm^−1^. Additionally, a decrease in the vibration intensity of O-Si-O bonds visible before immersion and the appearance after immersion of a new absorption band at 440 cm^−1^, prove the presence of phosphate groups in the samples’ structure [49].

The very slow rate of apatite formation on the surface of forsterite after immersion in SBF for 14 days is due to the presence of magnesium content in the material. This formation process of bone-like apatite on the surface of silicate bioceramics in SBF continues until the complete crystallization of amorphous apatite at the surface is reached by consuming other essential ions (OH, CO_3_^2−^) from the SBF [49].

**Figure 15:**
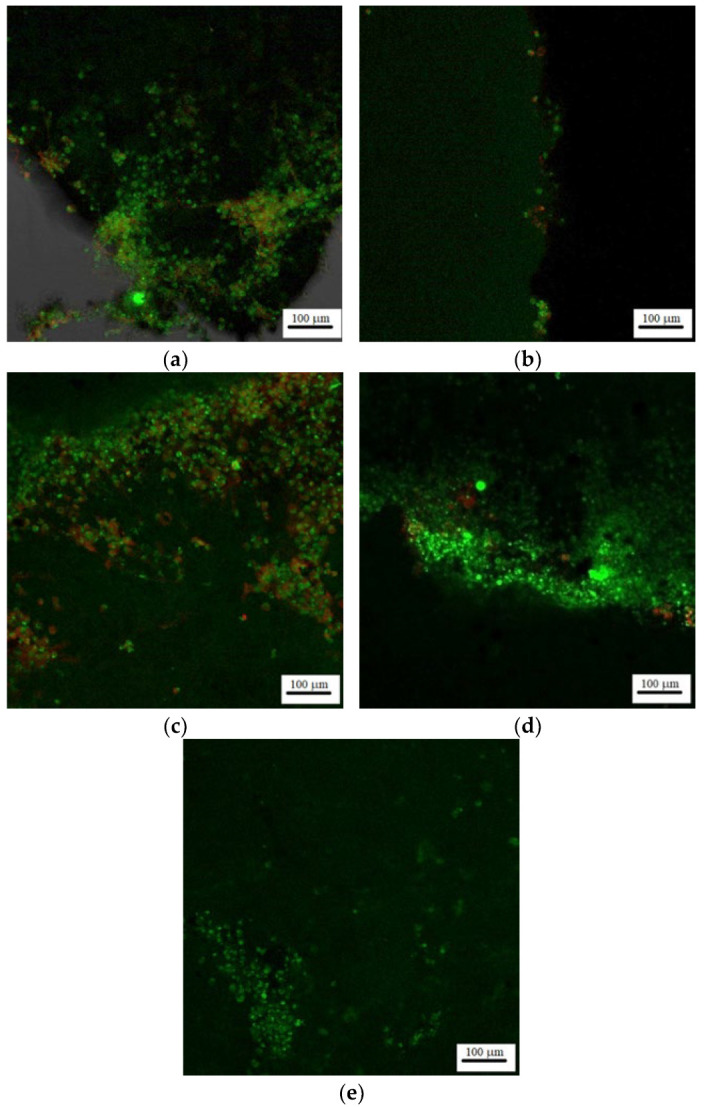
Florescence microscopy for images for F_1_ (**a**), F_1_20s (**b**), F_2_ (**c**), F_2_20s (**d**) and control (**e**) samples.

According to fluorescence microscopy, the cells responded to the contact with forsterite samples treated at different temperatures and different architectures can be seen. Thus, a slight attempt of cell adhesion to the substrate of the sample can be seen in Figure 15 specific to sample F_1_. This sample, compared to the F_1_20s sample (Figure 15b), is more promising in terms of cell adhesion.

The cells appear to agglomerate more easily to the surface of the sample treated at 1320 °C. The F_2_20s sample (Figure 15d) shows the best cell viability of all forsterite samples. The cells adhere well to the environment. It was found that this sample was able to stimulate cell growth. The good cell behaviour at the material interface may be due to the microporous architecture induced by the addition of the porogenic agent.

## 4. Conclusions

The present study aims to develop ceramics with controlled porosity by adding a porogenic agent and varying the sintering treatment. The obtained materials were characterized from morpho-structural and mechanical points of view, as well as by assessing the in vitro biocompatibility. The porogenic agent influenced the sample porosity, without altering the morphology of the forsterite crystals or the particle size. Moreover, it was efficiently removed during the heat treatment. The effect of the porogenic agent no longer seems significant after sintering at 1250 °C, and the difference in porosity between the two thermally treated ceramics appears negligible. Both temperatures used in this study determined the formation of the crystalline phase, as observed when applying XRD to the forsterite phase. The presence of the enstatite (MgSiO_3_) as secondary phase in small quantities can also be highlighted, with the diffraction maxima being very low in intensity. The compressive strength resistance was well-balanced with the number and volume of the generated pores; the registered values were close to the cortical bone tissue theoretical value. The samples were able to stimulate cell growth, especially in the case of scaffolds with a microporous architecture induced by the addition of sucrose. The obtained results seem promising and constitute the basis of extensive study regarding the osteoinduction and osteoconduction of the acquired porous ceramics.

## Figures and Tables

**Table 1:** Sample formulation and thermal conditions for obtaining ceramic materials.

Sample Name	Sintering Temperature	Porogenic Agent (% wt.)
F_1_	1250 °C	0
F_2_	1320 °C	
F_1_20s	1250 °C	20
F_2_20s	1320 °C	

**Table 2:** Chemical composition of samples after 14 days in SBF.

Elements	F_1_		F_2_		F_1_20s		F_2_20s	
	Weight %	Atomic %	Weight %	Atomic %	Weight %	Atomic %	Weight %	Atomic %
**O**	46.48	58.35	47.5	59.06	45.83	57.51	47.47	59.04
**Mg**	33.84	27.96	34.89	28.55	35.33	29.17	34.68	28.38
**Si**	16.87	12.07	17.07	12.09	17.93	12.82	17.38	12.31
**Ca**	1.53	0.99	0.26	0.17	0.31	0.2	0.22	0.14
**P**	1.27	0.64	0.28	0.14	0.59	0.3	0.25	0.13

## Data Availability

All the data is available within the manuscript.
